# Stress Proteins and Pancreatic Cancer Metastasis

**DOI:** 10.1100/tsw.2010.186

**Published:** 2010-10-01

**Authors:** Carla E. Cano, Juan L. Iovanna

**Affiliations:** INSERM U.624, Stress Cellulaire, Parc Scientifique et Technologique de Luminy, Marseille, France

**Keywords:** cancer, pancreas, cell stress response, Nupr1, metastasis

## Abstract

Tumor metastasis is challenged by its resistance to microenvironmental stress infringed during escape from the primary tumor and the colonization of a foreign secondary tissue. Because of its great metastatic potential and its strong resistance to anticancer drugs, pancreatic cancer is regarded as a paradigm of the adaptation of cancer cells to microenvironmental stress. Thus, to understand how pancreatic cancer cells adapt to the different endogenous and therapy-related stresses is crucial for understanding their etiology and for the development of new efficient anticancer strategies. This review summarizes the multiple functions accomplished by one major factor of pancreatic cancer cell stress response, the stress protein p8.

## INTRODUCTION

Metastasis is the main cause of cancer death. This observation is particularly true in the case of pancreatic cancer. As a matter of fact, more than 85% of patients with pancreatic cancer presents with metastasis at diagnosis. In this setting, the disease is virtually untreatable since resective surgery, which is the only curative treatment available, becomes counterindicated. The picture turns even darker when one considers the remarkably high resistance of pancreatic cancer cells to radio- and chemotherapies. Since pancreatic cancer represents the fourth leading cause of cancer death in western countries, understanding the mechanisms that allow such a rapid and efficient metastasis formation in this malignancy becomes mandatory for the development of new and more efficient treatments of this disease. Pancreatic ductal adenocarcinoma is the more abundant and aggressive form of pancreatic cancer and, therefore, will be the focus of this manuscript. In pancreatic adenocarcinoma, as in most cancers, metastasis formation is conditioned by the capacity of cancer cells to invade foreign tissues and to prosper within their new environment, often very hostile. Therefore, aggressiveness of cancer cells depends on their ability to survive in the blood flow and to adapt to the invaded tissue. Cancer cells that evade from the primary tumor will not form metastasis in every tissue. In the case of pancreatic adenocarcinoma, the liver is the most frequent target, although it could also spread to the bowel, the lungs, the bone, and the adrenal glands.

To target pancreatic cancer metastasis efficiently, the general and tissue-specific characteristics of this pathology must be considered. Metastasis is a selective process that depends on both the interactions between the cancer cell and the target organ, and the environmental factors to which the cancer cell will be exposed (Fig. 1)[1,2,3,4]. In fact, cancer develops through sequential steps. First, within the primary tumor, cancer cells are selected on the basis of their fast growth, their resistance to apoptotic signals, and their ability to escape to the antitumoral immune reaction. Then, tumor cells leave the primary tumor and “travel” through the blood flow. Ever since, cancer cells must display the skills necessary to invade the target organ that, most of the time, are not innate skills of the original cancer cells. Finally, and we come to our point of interest, cancer cells must be able to settle within the target organ, to grow, and to form a secondary tumor or metastasis. Disruption of the invaded tissue comes as a collateral damage.

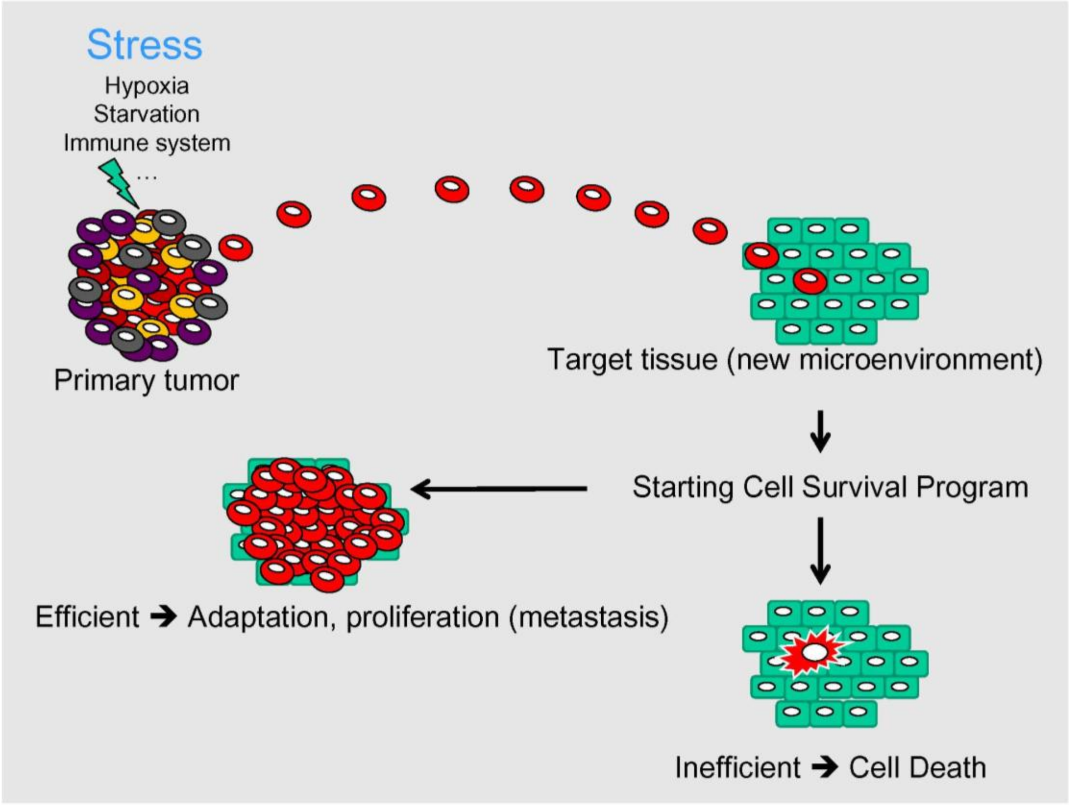

**FIGURE 1.** Model of microenvironmental stress-driven selection of metastatic cancer cells. Metastasis implantation and growth is preceded by at least two waves of cancer cell selection. The first wave is triggered within the primary tumor as malignant cells disrupt their original tissue and grow away from the vessels, rapidly becoming starved of nutrients and oxygen. As a response to these adverse conditions, cancer cells launch a transcriptional response. Only those cells whose gene expression profile confers efficient resistance to starvation, apoptosis, poor immunogenicity, and migratory skills will be allowed to leave the primary tissue and to travel through the blood stream. The second selection wave is triggered by the specific features of the invaded organ. Similar to the first wave, the genetic response is established in cancer cells in order to adapt and to grow within the invaded tissue. Failure to establish a suitable genetic profile (and the related phenotype) in response to stress will lead to deletion of the cancer cell.

The phenotype of the original cancer cell is fashioned by the physiology of the organ where the primary tumor develops. Because the environment of the target tissue is inevitably different from the one within the primary tumor, the colonization of this new tissue will cause a concomitant stress to the cancer cell, and the ability to overcome this stress will condition cancer cell survival and implantation. Invasive cancer cells must therefore activate a genetic defense program to ensure survival and to be able to proliferate and to form a metastasis. It is likely that, for a given tissue, this genetic program follows a similar pattern to a stress response program displayed during common pathologic disorders affecting this tissue. Based on this hypothesis, we can use the current knowledge on pancreatic pathology to predict pathways that potentially participate in pancreatic cancer metastasis.

The most common illness of the exocrine pancreas is acute pancreatitis. In most cases, acute pancreatitis is a self-limiting disease, owing to the ability of pancreatic cells to hold back the injury by switching on a genetic "emergency program". We have previously demonstrated that this program, known as the pancreatic stress response, is characterized by a dramatic increase of gene expression and protein synthesis in the acinar cells[5], with a selective down-regulation of genes encoding potentially harmful enzymes (i.e., amylase and chymotrypsinogen) and a strong up-regulation of other genes encoding presumably protective factors or stress response genes. Most of the overexpressed stress genes identified were already acknowledged to prevent the evolution of pancreatitis (i.e., pancreatitis-associated protein *PAP*, lithostathine), or as factors of pancreatic regeneration following pancreatitis (i.e., c-myc, H-ras, and clusterin)[6]. Furthermore, large-scale analysis of the acute pancreatitis–related gene expression profile led to the cloning and characterization of new stress genes. In the last years, a mass of data from our lab and others has revealed that several stress genes induced during acute pancreatitis play a role during tumor progression and metastasis formation. This review will focus on the pleiotropic protumoral functions of one pivotal factor of the pancreatic stress response, the stress protein p8.

## DISCOVERY OF THE STRESS PROTEIN P8/NUPR1

The Nupr1 gene, encoding the stress protein p8, was identified 13 years ago after its strong up-regulation during the acute phase response of pancreatic acinar cells to sodium taurocholate–induced pancreatitis in the rat[7]. Nupr1 is a widely conserved gene that has been cloned in mammals[7,8,9], *Xenopus*[10], *Drosophila*[11], zebrafish[12], shrimp[13], urchin[14], and nematode (GenBank accession ABLE02003691). No paralogue has been found, however, in yeast. In humans, p8 messenger RNA is 600 nucleotides long and encodes an 82-amino-acid-long polypeptide with a theoretical molecular mass of 88.72 kDa. The p8 protein contains a canonical bipartite domain of positively charged amino acids typical of nuclear-targeting signals (NLS) and a N-terminal PEST (Pro/Glu/Ser/Thr-rich) region, suggesting nuclear localization and regulation of p8 protein life span by the ubiquitin/proteasome system, which were experimentally confirmed[9,15]. Alternative splicing produces an isoform, named isoform α, that contains 18 additional amino acids and for which no function has been reported. Thus far, all of the data on p8 characterization concern the shorter isoform β. p8 isoforms α and β share similar secondary structures and *in silico* analysis predicts the presence of a basic helix-loop-helix motif characteristic of some classes of transcription factors. Biochemical properties of the mammalian p8 proteins include a high isoelectric point (9.6–10.4), 14% of acidic amino acids, 20–24% of basic amino acids, 14–17% of serine/threonine/tyrosines, 6–9% of praline, and 5–6% of glycine. The negatively charged residues appear located at the amino-terminal end, whereas all positively charged residues accumulate in the carboxyl-terminal region of the molecule[16].

## P8 IN TUMOR PROGRESSION AND METASTASIS

The p8 protein is overexpressed in several types of human cancers and, in particular, in late stages of pancreatic cancer and its metastasis[17,18,19,20]. This observation pointed to the hypothesis that p8 may play a promoting role during cancer progression since, as it is expected from a stress response factor, p8 could help the adaptation of cancer cells to stressful conditions on growing primary tumors and during the implantation within secondary organs. According to this hypothesis, Ree et al. found the Com1 gene, identical to Nupr1/p8, as being eightfold up-regulated during implantation of breast metastasis from disseminated breast cancer cells[21].

In order to formally establish the implication of p8 in cancer cell implantation within foreign tissues, we developed a genetically modified mouse bearing a deletion of the exon 2 of the Nupr1/p8 gene, which encodes 60% of the coding sequence[22]. These mice are viable and fertile, but present increased susceptibility to the pancreatic stress undergone during experimental acute pancreatitis[23]. We derived embryonic fibroblasts from p8-deficient mice that we transformed by retroviral transduction of the rasV12 and E1A oncogenes. As expected, the p8-wild type–transformed fibroblast formed tumors when injected subcutaneously or in the peritoneal cavity of nude mice. In contrast, the p8-deficient–transformed fibroblasts were unable to form either subcutaneous or intraperitoneal tumors in nude mice[24]. Conversely, restoration of p8 expression using a retroviral vector led to recovery of the tumor-forming capacity of p8-deficient rasV12/E1A fibroblasts. Knock-down of p8 expression in the aggressive pancreatic cancer cell line MiaPaCa-2 yielded the same results in terms of tumor formation in athymic mice (our unpublished data). These results clearly demonstrate that p8 expression is necessary for cancer cell implantation in a foreign tissue and encouraged us to perform further research in order to determine the mechanisms underlying the cancer-promoting action of p8.

## REGULATION OF P8 EXPRESSION UPON STRESS

*In vivo*, different cell stress agents were shown to activate p8 transcription. For instance, p8 expression is induced in the pancreas, liver, and kidney after systemic injection of lipopolysaccharide[25]. Moreover, the Nupr1 gene appears to be a good sensor of even very mild stresses, since p8 synthesis is activated upon a simple culture medium replacement[26]. Very interestingly, p8 expression is activated in response to most common aggressions undergone by cancer cells during cancer progression, including growth inhibitory signals[27,28], starvation[9], hypoxia (our unpublished results), apoptosis inducers, and anticancer drugs[9,29].

The cloning and analysis of the Nupr1/p8 promoter shed light on the regulation of p8 expression in cancer. Several specific target elements of key transcription factors implicated in cancer progression are present in this promoter. The first transcription factors described to regulate p8 transcription through a CAAT-C/EBP cis-acting element were C/EBPα and C/EBPβ[8]. In addition to their multiple functions during specialized cell differentiation and tumor progression[30,31,32], C/EBPβ has recently been implicated in intravascular survival of pancreatic cancer cells[33]. Furthermore, the Nupr1/p8 promoter contains an NF-κB–specific motif that is activated by NF-κB binding upon tumor necrosis factor challenge[34], suggesting a possible role of p8 in the NF-κB pathway. It is needless to argue the prosurvival and proinflammatory roles of the NF-κB pathway in pancreatic cancer, which have been thoroughly summarized elsewhere[35]. Finally, the Nupr1/p8 promoter bears three Smad-responsive elements (SRE) whose function was experimentally confirmed[28]. This is of particular interest to pancreatic adenocarcinoma since the Smad complex is composed, among other Smad proteins, by the Smad4/DPC4 tumor suppressor that is mutated in around 50% of human pancreatic cancers.

## ROLE OF P8 DURING THE CELL RESPONSE TO TGFB

The transforming growth factor beta (TGFβ) is a secreted polypeptide belonging to a wide family of cytokines and growth factors, including TGFβs, bone morphogenetic proteins (BMPs), and activins. TGFβ signaling involves receptors composed of two subunits, TβRI and TβRII (type I and II TGFβ receptors). After binding to its receptors, TGFβ induces the phosphorylation of TβRI, which phosphorylates receptor-associated Smad2 and 3 (R-Smads). Phosphorylated Smad2 and 3 interact with Smad4/DPC4. The Smad2, 3, and 4 complexes accumulate within the nucleus, bind to DNA, and activate the transcription of target genes through binding of the SREs. In addition to the “canonical” Smad signaling pathway, TGFβ activates Smad-independent pathways (MAPK, PI3K/Akt). TGFβ controls a number of fundamental cell processes, including proliferation, migration, adhesion, differentiation, and extracellular matrix remodeling. In cancer, TGFβ plays a double-edged sword role, for it induces cell cycle arrest and apoptosis during early tumorigenesis but, once the malignant cell overcomes these cytostatic effects, it favors metastasis by inducing the epithelial-to-mesenchymal transition (EMT). During this transition, epithelial cancer cells acquire a more aggressive fibroblastic phenotype with higher motility, migration, and proteolytic properties, which are essential for extravasation and metastasis. Factors that mediate the switch between the tumor-promoting and -suppressing effects of TGFβ are currently unknown. Since p8 is both a TGFβ-inducible and a tumor-promoting factor, it is possible that p8 may be a switch factor toward TGFβ protumoral function. To test this hypothesis, we first determined the p8 functional relationship with the Smad complex, which seems to be implicated in both branches of cancer-associated TGFβ functions in the pancreas. Indeed, Smad4/DPC4 deletion favors development of pancreatic tumors in mice bearing a constitutively activated K-ras$^{G12D}$ allele targeted to the pancreas[36]. Nevertheless, Smad4 deficiency provokes an EMT blockade in pancreatic adenocarcinomas developed in K-ras$^{G12D}$p16KO mice. In order to establish the role of p8 in TGFβ signaling, we assayed its implication in the Smads-transcriptional activity using mouse embryonic fibroblasts (MEFs) derived from our p8-deficient mice. As expected, Smad-mediated promoter activation was lower in p8-deficient MEFs compared to wild type. Restoration of p8 expression in deficient cells using a human p8-encoding plasmid restored Smad-mediated transcription[28]. We therefore concluded that p8 is necessary for Smad-induced transcription upon TGFβ stimulation. Accordingly, the growth inhibitory effect of TGFβ in the Smad-proficient human pancreatic cell line Panc-1 is abolished by inactivation of p8 expression using a specific antisense RNA[37]. In addition, p8 knock-down in these cells leads to alterations of EMT-related gene expression upon TGFβ treatment (our unpublished data). Moreover, we also observed alterations of TGFβ-induced gene expression by p8 inactivation in Smad-deficient cell lines, thus suggesting that p8 plays a role in TGFβ-induced EMT in both Smad-wild type and -mutated pancreatic tumors. Further research is needed to formally establish the implication of p8 in TGFβ-induced EMT in pancreas cancer cells.

## P8 AND CELL CYCLE REGULATION

Regulation of cell cycle checkpoints upon cell injury is one of the best-characterized functions of the cell stress response. Therefore, it was not surprising to find that p8 is implicated in the control of cell cycle progression. As a matter of fact, p8-deficient MEFs present increased proliferation and incorporation of [3H] thymidine and BrdU than wild-type fibroblasts[38]. Moreover, p8-deficient MEFs show resistance to cell cycle arrest induced by serum deprivation, indicating a pivotal role for p8 during cell cycle checkpoints. Increased cyclin-dependent kinases (Cdk)-2 and Cdk4 activities in p8-deficient cells compared to wild type indicated that p8 acts upstream of the Cdk-mediated checkpoint. Indeed, we found that p8 regulates the nucleus-to-cytoplasm translocation and subsequent degradation of the p27 cdk-inhibitor[39]. This is mediated by p8 protein-protein interaction with the regulatory factor Jab1, a component of the COP9 signalosome complex. Moreover, p8 interacts with p53, an interaction that allows the expression of another cdk-inhibitor, p21(Waf1/Cip1)[40]. Therefore, it appears that p8 favors cell recovery from an eventual stress by mediating cell cycle arrest through inhibition of cdk activities.

## P8 AND RESISTANCE OF PANCREATIC CANCER CELLS TO APOPTOSIS

In human pancreatic cancer, high levels of p8 protein expression correlate with low apoptosis[17]. This inverse correlation is remarkable in pancreatic cancer metastasis, suggesting that p8 overexpression counteracts apoptosis-inducing signals associated with colonization of secondary tissues. We previously demonstrated that p8 inhibits apoptosis by its interaction with one well-known antiapoptotic partner, the prothymosin-α (ProTα)[41]. ProTα is an acidic protein that shares with p8 the characteristic of having an unstable tertiary structure. ProTα is an acknowledged inhibitor of mitochondria-dependent apoptosis by preventing apoptosome formation and subsequent caspase-9 activation[42]. Using biophysical approaches (circular dichroism and nuclear magnetic resonance), we demonstrated that p8 and ProTα form a stable heterodimeric complex, allowing stabilization of their tertiary structure. Knock-down and converse overexpression experiments allowed us to demonstrate that the interaction with p8 is required for the antiapoptotic function of ProTα[41]. In other words, ProTα antiapoptotic function is actually mediated by the p8/ProTα complex[41]. Inactivation of either partner leads to apoptosis. Therefore, the complex p8/ProTα represents a new promising antiapoptotic target for pancreatic cancer treatment. The interaction between p8 and ProTα may explain, at least partially, the inability of p8-deficient xenografts to form tumors in athymic mice. In addition, the antiapoptotic function of p8 is involved in the resistance of pancreatic cancer cells to the most common drug used to treat this cancer, gemcitabine[43]. Indeed, we have observed that p8 expression levels are directly proportional to resistance to gemcitabine and, conversely, inactivation of p8 expression in resistant cells strongly increases cell sensitivity to this drug. Furthermore, overexpression of p8 in gemcitabine-sensitive cells increases their survival upon gemcitabine treatment. Altogether, our findings show that p8 expression significantly increases the resistance of pancreatic cancer cells to apoptosis.

## P8 ACTION ON CHROMATIN REMODELING

The HMG-like biochemical features and DNA-binding ability of p8 suggest that the action of this stress protein in cancer may involve regulation of gene expression in response to tumor microenvironment stresses. In addition, the diverse panel of functions accomplished by p8 indicates that it could affect stress-related gene expression on a large scale. In this sense, we have recently demonstrated that p8 interacts and regulates the activity of the Male-Specific Lethal (MSL)-1 protein that is involved in chromatin remodeling[44]. MSL1 is a nuclear protein that was first identified in *Drosophila* for its implication in the MSL complex responsible for the dosage compensation of X-chromosome transcription[45]. Both in flies and humans, the MSL complex acetylates the lysine 16 of the histone H4 and induces chromatin accessibility and gene transcription activation[46]. Therefore, the interaction with MSL1 opens the possibility of a role of p8 in the regulation of chromatin accessibility. We confirmed the involvement of p8 in the MSL complex by coimmunoprecipitation of the p8/MSL1 heterodimer with the MORF4L1 and MRG15 proteins, two other members of this complex. Moreover, we showed that p8 overexpression, together with MSL1 interaction, inhibits H4-lysine 16 acetylation[44]. Conversely, we found alterations of HDAC activity in p8-deficient cells. Therefore, the p8/MSL1 may orchestrate the accessibility and activation of many gene promoters in cancer cells upon a given stress. p8 involvement on the establishment of the “chromatin landscape” in response to stress could explain the spectacular changes in gene expression that we have observed after p8 inactivation in stressed cells.

Furthermore, we and others have shown that p8 possesses a cotranscription factor activity through its interaction with p53 and p300[40,47]. The p8/p300 complex interacts with PTIP (Pax2 transactivation domain-interacting protein), thereby derepressing gene transcription[47]. In addition, p8 has also been shown to coordinate p300, p68/Ddx, and MyoD binding to myogenin promoter in myocytes[48].

## CONCLUSION

The recalcitrant resistance of pancreatic cancer to all available anticancer therapies vindicates its uniqueness among other carcinomas. Therefore, the development of new efficient strategies to fight pancreatic cancer metastasis needs to take into account the nature of pancreatic cells and their innate ability to endure adverse environmental conditions. The stress protein p8 is a small chromatin protein that provides a shield to normal and malignant pancreatic cells against stress. Its expression is induced following even very mild assaults. p8-regulated cell processes include transcription, cell cycle checkpoint, TGFβ-signal transduction, and apoptosis. Since p8 action confers a survival advantage to cancer cells upon stress, it appears as a promising drug-targetable stress protein for the treatment of pancreatic cancer with a predictable effect in metastasis formation. Because the pleiotropic functions of p8 are mediated by its interaction with various molecular partners, molecules that block the formation of p8-containing complexes may act as efficient anticancer drugs that affect precise p8 functions with limited side effects.
